# ELISA-Based Enzyme Kinetics Assay for Measuring cGAS Activity

**DOI:** 10.21769/BioProtoc.5655

**Published:** 2026-04-05

**Authors:** Cécile Fréreux, Philip H. Howe

**Affiliations:** Department of Biochemistry and Molecular Biology, Medical University of South Carolina, Charleston, SC, USA

**Keywords:** cGAS, Enzyme kinetics, 2′3′-cGAMP synthesis, ELISA, Accessible, Michaelis–Menten, V_max_, k_1/2_, k_cat_

## Abstract

Cyclic GMP–AMP synthase (cGAS) is a key cytosolic double-stranded DNA sensor that activates innate immune responses. Upon binding double-stranded DNA, cGAS undergoes conformational activation and catalyzes the synthesis of the second messenger 2′3′-cyclic GMP–AMP (2′3′-cGAMP) from ATP and GTP. 2′3′-cGAMP then triggers a downstream signaling cascade that induces type-I interferon and inflammatory gene expression and has been shown to exert antitumor effects in the context of cancer. Accurate measurement of this enzymatic activity is therefore important for mechanistic studies. Traditional kinetic methods such as radiolabeling, HPLC, or mass spectrometry provide precise results but require specialized equipment and expertise. Here, we describe a rapid and accessible ELISA-based protocol to quantify 2′3′-cGAMP product formation and derive cGAS enzymatic parameters. Reactions are initiated with defined DNA ligands and quenched at multiple time points, and product accumulation is quantified by a commercially available 2′3′-cGAMP ELISA. Time course measurements are used to calculate initial velocities, which can be plotted against substrate concentration to obtain Michaelis–Menten parameters. This approach enables direct, product-specific quantification of 2′3′-cGAMP formation using only an absorbance plate reader. The protocol provides a sensitive and broadly applicable alternative to traditional methods, allowing laboratories without advanced instrumentation to perform reliable cGAS enzyme kinetics.

Key features

• Developed to evaluate how cofactors or regulatory proteins modulate cGAS activity in vitro.

• Suitable for assessing cGAS kinetics with diverse nucleic acid substrates under defined reaction conditions.

• Determination of initial (V_0_) and maximal (V_max_) reaction rates, catalytic turnover number (k_cat_), and k_1/2_, the DNA concentration at which cGAS reaches 1/2 V_max_.

• Yields reproducible kinetic results within ~4 h from reaction setup to data analysis.

## Graphical overview


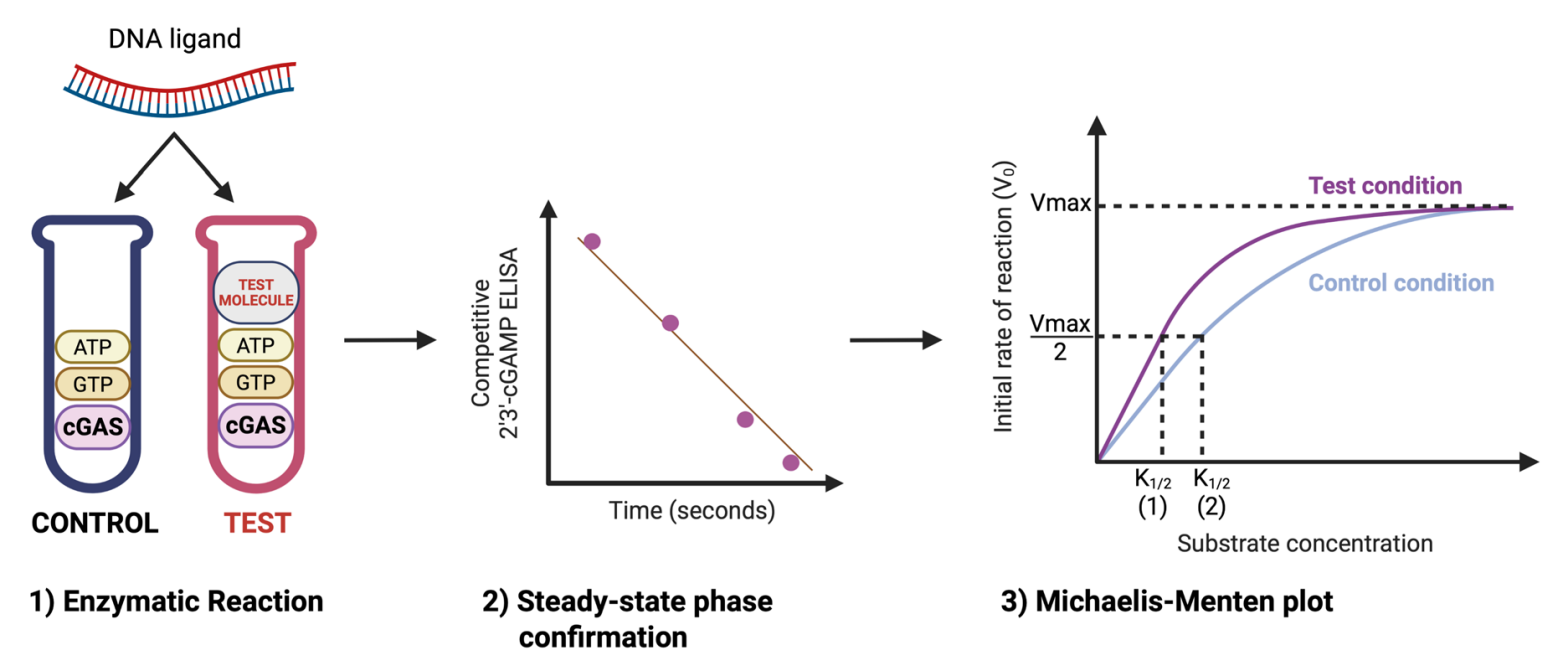


## Background

Cyclic GMP–AMP synthase (cGAS) is a cytosolic DNA sensor that catalyzes the synthesis of the second messenger 2′3′- cGAMP from ATP and GTP. Activation of cGAS triggers the STING pathway, leading to type-I interferon production and downstream immune responses. Because of its central role in innate immunity, autoimmunity, infection, and cancer biology [1], there is strong interest in understanding how cGAS activity is regulated and how cofactors, binding partners, or small molecules influence its enzymatic function.

Several methods have been described to measure cGAS activity. The most direct approaches rely on HPLC, LC–MS/MS, or radiolabeled nucleotide assays, which allow high sensitivity and precise quantification of 2′3′-cGAMP but require specialized instrumentation, radioisotope handling, or technically demanding protocols [2–5]. More accessible alternatives use ELISA-based detection kits that exploit the high specificity of antibodies against 2′3′-cGAMP. ELISAs offer simplicity, scalability, and compatibility with standard laboratory equipment, making them well-suited for kinetic analyses in laboratories without access to advanced analytical platforms.

The protocol described here provides a streamlined workflow to determine Michaelis–Menten kinetic parameters (V_max_, k_cat_, k_1/2_) [6,7] of cGAS using an endpoint ELISA readout. Compared to continuous real-time methods, this approach requires only standard molecular biology equipment and commercially available reagents, making it broadly applicable. While endpoint ELISA measurements rely on the assumption of linear product accumulation within the chosen incubation window, this can be validated with short-time-course experiments. Once established, the protocol enables reproducible assessment of how test molecules (e.g., protein cofactors or small molecules) alter cGAS catalytic activity and substrate affinity.

Beyond evaluating candidate regulators of cGAS, the protocol can be adapted to study mutant cGAS variants, viral or cellular proteins that interact with cGAS, or pharmacological inhibitors and activators. Together, this method provides an accessible and adaptable framework for interrogating DNA-sensing enzymatic activity and for identifying potential therapeutic modulators of innate immune signaling.

## Materials and reagents

**Reagents**

1. Nuclease-free, molecular biology–grade water (Thermo Scientific, catalog number: J71786.XCR)

2. Tris-HCl pH 7.5 (1 M) (Fisher BioReagents, catalog number: BP152-5)

3. MgCl_2_ (1 M) (Sigma-Aldrich, catalog number: 208337)

4. Tween-20 (10%) (Fisher BioReagents, catalog number: BP337-500)

5. Adenosine triphosphate (ATP), 5 mM (Cayman Chemical, catalog number: 40182); store in aliquots at -20 °C

6. Guanosine triphosphate (GTP), 5 mM (Cayman Chemical, catalog number: 16060); store in aliquots at -20 °C

7. cGAS DNA ligand, forward and reverse sequences ordered from Eurofins [8]

8. His-cGAS human recombinant protein (10 μM, activity 3.10 U/mL) (Cayman Chemical, catalog number: 22810)

*Note: The enzyme is extremely temperature sensitive. Store in ~5 μL aliquots at -80 °C. Use each aliquot only once to ensure the best reproducibility.*

9. Ethylenediaminetetraacetic acid (EDTA), 55 mM (Sigma-Aldrich, catalog number: ED-100G)

10. 2′3′-cGAMP ELISA kit (Cayman Chemical, catalog number: 501700); store at 4 °C

**Solutions**

1. Reaction buffer 4× (see Recipes)

2. Recombinant cGAS protein dilution 1/10 (see Recipes)

**Recipes**

**1. Reaction buffer 4×**

**Table:** 

Reagent	Quantity or volume	Final concentration
Tris-HCl, pH 7.5 (1 M)	200 μL	40 mM
MgCl_2_ (1 M)	50 μL	10 mM
Tween-20 (10%)	10 μL	0.02%
Nuclease-free water	4.74 mL	
Total	5 mL	

Prepare fresh before the assay or store at 4 °C for up to 3 months. Alternatively, aliquot and store at -20 °C for up to 6 months.

**2. Recombinant cGAS protein dilution 1/10**

**Table:** 

Reagent	Quantity or volume	Final concentration
His-cGAS enzyme (10 μM stock)	20 μL	1 μM
Reaction buffer (4×)	50 μL	1×
Nuclease-free water	130 μL	
Total	200 μL	

The recombinant His-cGAS protein was provided by the manufacturer at 10 μM in 50 mM HEPES, pH 8.0, with 300 mM sodium chloride and 10% glycerol. Concentration may slightly vary between production batches.

Dilute the cGAS enzyme in reaction buffer as shown (example for 200 μL of final volume, sufficient for 40 reactions).

*Note: The dilution can be performed immediately before the kinetic experiment. The diluted enzyme may be aliquoted (~5 μL) and stored at -80 °C for up to 1 month, as stability is reduced after 1:10 dilution. Use each aliquot only once. Do not subject aliquots to repeated freeze-thaw cycles.*

**Laboratory supplies**

1. 0.2 mL PCR tubes (Thermo Scientific, catalog number: AB0620)

## Equipment

1. T100 thermal cycler (Bio-Rad, catalog number: 1861096)

*Note: Preferably, use a thermocycler for precise temperature control. Alternatively, a calibrated dry heat block or water bath set to 37 °C may be used. The enzyme reactions can also be performed at room temperature, but that may reduce the efficiency of the enzyme.*

2. SpectraMax iD5 microplate reader (Molecular Devices, model: ID5-STD) or any plate reader able to read the absorbance of 96-well plates at 450 nm

3. Multichannel pipette (1–10 μL) (Thermo Scientific, catalog number: 4661000N)

## Software and datasets

1. GraphPad Prism (Version 10.5.0)

## Procedure

**A. Overview of kinetic assay design**

This protocol allows investigators to test the effects of a molecule on cGAS enzymatic activity. From the determination of V_max_ and k_cat_, one can assess whether the molecule alters the intrinsic catalytic activity of cGAS, while the determination of k_1/2_ indicates whether the molecule influences the apparent affinity of cGAS for its DNA ligand.

The substrates of cGAS are ATP and GTP, which are catalyzed to form 2′3′-cGAMP. However, cGAS requires binding to a DNA ligand as an allosteric activator to adopt the correct conformation for ATP and GTP binding and subsequent catalysis. For this reason, the DNA ligand concentration is varied and plotted on the X-axis to generate the Michaelis–Menten curve [6,7].

The following procedure illustrates how to set up a single enzymatic reaction in parallel test and control tubes: a test condition containing the molecule of interest and a control condition without it, at a defined reaction time and DNA ligand concentration. To derive kinetic parameters (V_max_, k_cat_, k_1/2_), multiple reactions must be performed, varying both the incubation time and the DNA ligand concentration.

As a first step, perform short-time-course experiments for both test and control conditions (e.g., 30 s, 2 min, 5 min) at 10 nM DNA and 500 nM DNA (saturating concentration), with 30 nM cGAS and 100 μM each of ATP and GTP in the reaction. This ensures that product accumulation is linear with time, which is critical because only in the linear range can endpoint measurements be used to reliably reflect the initial velocity (V_0_). This linearity should be confirmed under the user’s specific conditions, but it has been validated with the following protocol under the conditions described here.

Once linearity is confirmed, select a fixed incubation time (we recommend 30 s under these conditions). Then, perform the assay across a range of DNA ligand concentrations under both test and control conditions (e.g., 2.5, 5, 7.5, 15, 50, and 500 nM). Plotting initial velocity (V_0_, expressed as nM 2′3′-cGAMP produced per minute) vs. DNA concentration will generate the Michaelis–Menten curves from which V_max_, k_1/2_, and k_cat_ can be calculated in the presence and absence of the test molecule. These parameters enable direct interpretation of the molecule’s effect on cGAS activity.

**B. Enzymatic reaction**

1. Anneal the cGAS DNA ligand by mixing forward and reverse oligonucleotides at a 1:1 molar ratio, each at 100 μM. Heat the mixture in a beaker of boiling water maintained under stirring to fully denature the DNA, then allow it to cool gradually to room temperature over at least 5 h or overnight to ensure complete annealing. The resulting double-stranded DNA has a final concentration of 50 μM and is further diluted with nuclease-free water for use in the reactions in step B6.

2. Pre-equilibrate all reagents to room temperature, except for recombinant proteins (cGAS, or other potential cofactors to test).

3. Pre-heat a thermocycler or any other tube incubator at 37 °C.

4. Prepare quench tubes by dispensing 8 μL of 55 mM EDTA into 0.2 mL PCR tubes (one tube per enzymatic reaction). 5 μL from each EDTA tube will be used at a later step to stop all enzymatic reactions simultaneously using a multichannel pipette (step B10).

5. Prepare the reaction master mix: In a 0.2 mL PCR tube, combine the following (example for 2 reactions = control + test; scale proportionally for additional reactions): 19.8 μL of nuclease-free water, 27.5 μL of reaction buffer 4× (2× final concentration), 2.2 μL of ATP 5 mM (200 μM final concentration), 2.2 μL of GTP 5 mM (200 μM final concentration), and 3.3 μL of recombinant His-cGAS 1 μM (60 nM final concentration). Total volume is 55 μL.

**Critical:** Thaw a single 5 μL aliquot of recombinant His-cGAS immediately before use and add it as the final component to the master mix. Do not refreeze; discard any remaining enzyme after use. Prolonged exposure of the enzyme to room temperature or repeated freeze-thaw cycles reduces activity.

6. Prepare two 0.2 mL PCR tubes for your control and test condition containing the following volumes of reaction master mix, test molecule, nuclease-free water, and DNA ligand. It is important to add the DNA ligand last to both tubes simultaneously using a multichannel pipette, as this will start the enzymatic reaction (t_0_).

*Note: This reaction will have to be performed with varying amounts of DNA ligands to plot a Michaelis–Menten curve. See Table 1 for the suggested amounts of DNA ligand.*

**Table 1.** Reaction setup for 2′3′-cGAMP synthesis assays under control and test conditions

	Control condition	Test condition
Reaction master mix (step B6)	25 μL	25 μL
Test molecule	NA	To optimize, e.g., 3 μL of test protein at 1 μM (= 60 nM final concentration)
DNA ligand 0.5 μm stock variable (add last)	1.5 μL	1.5 μL (15 nM final concentration)
Nuclease-free water	Volume for 50 μL	Volume for 50 μL
Total	50 μL	50 μL

*Note: The final enzymatic reaction contains 100 μM of ATP, 100 μM of GTP, 10 mM Tris-HCl pH 7.5, 2.5 mM MgCl_2_, 0.005% Tween-20, 30 nM of recombinant His-cGAS, and variable amounts of test molecule (here, an example with 60 nM of test protein) and DNA ligand (here, an example with 15 nM, but it will be variable across enzymatic reactions).*

7. Immediately flick the tubes and briefly centrifuge in a tabletop PCR tube centrifuge.

8. Quickly place tubes in a thermocycler or tube-incubator pre-heated at 37 °C.

*Note: Alternatively, the reaction can be performed at room temperature, but it may reduce enzymatic activity. Keep the temperature consistent between replicates.*

9. Incubate for the desired amount of time.

*Note: Incubation between 30 s and 2 min typically yields 2′3′-cGAMP concentrations within the linear range of the ELISA standard curve, avoiding the need for sample dilution.*

10. Using a multichannel pipette, add 5 μL of 55 mM EDTA to each 50 μL reaction immediately after incubation to quench the reaction. Mix by flicking the tubes and briefly centrifuge. The final EDTA concentration is 5 mM, and the final volume per tube is now 55 μL.

*Note: Add EDTA immediately at the end of the incubation period to stop the reaction consistently across samples and ensure reproducible replicates.*

**Pause point:** Samples can be stored at -20 °C short-term or proceed directly with the ELISA.

11. Perform the enzymatic reaction steps B1–10 at several different time points (e.g., 30 s, 1 min, 1:30 min, 2 min) with a lower (e.g., 10 nM) and higher DNA ligand concentration (500 nM) to confirm linearity of product formation (see section A of Data analysis for an example of the linearity curve).

12. After confirming the linearity of product formation, choose a time within the window tested (e.g., 30 s) and perform the same enzymatic reactions at different DNA ligand concentrations (e.g., 2.5, 5, 7.5, 15, and 500 nM) to generate the Michaelis–Menten plot (see section B of Data analysis).

*Note: It is recommended to perform each enzymatic reaction at least twice, ideally three times, and independently to generate the Michaelis–Menten curve with good confidence intervals.*

**C. 2′3′-cGAMP ELISA**

Follow the manufacturer’s instructions for the 2′3′-cGAMP ELISA. Load 50 μL of each enzymatic reaction per well. The kit protocol allows incubation for 2 h at room temperature or overnight at 4 °C; we performed all incubations for 2 h at room temperature, which yielded consistent results.

*Note: Under the conditions described here, samples did not require dilution to perform the ELISA.*

## Data analysis

**A. Confirm linearity of 2′3′-cGAMP production at early time points**

**Figure 1.**
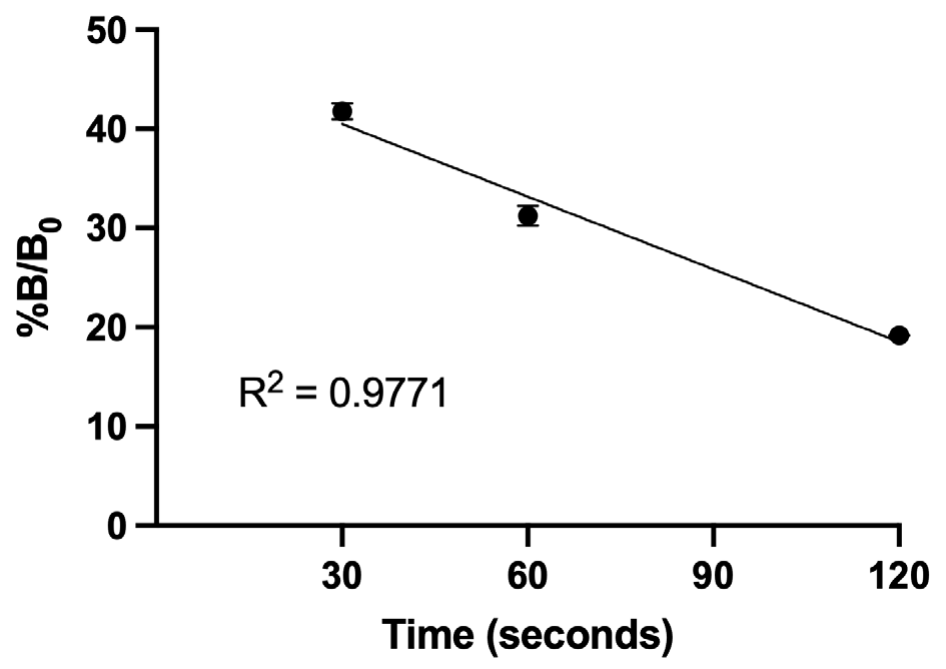
Linearity of 2′3′-cGAMP production at different time points. Reactions were performed with 30 nM recombinant cGAS and 500 nM DNA ligand (saturating conditions). Product accumulation was quantified by competitive ELISA at the indicated time points. The amount of 2′3′-cGAMP increased linearly with time (R^2^ = 0.9771), confirming that the chosen incubation window reflects the initial velocity (V_0_) under saturating substrate conditions. Data represent mean ± SD (n = 3) [8].

1. Plot the %B/B_0_ over time (from step B11) to confirm linearity of product formation. The R^2^ value should be close to 1 (Figure 1). In this competitive ELISA, B_0_ corresponds to the maximum binding signal observed in the absence of free 2′3′-cGAMP, when all antibodies are available to bind the 2′3′cGAMP tracer. B represents the signal from a given sample with an unknown amount of free 2′3′cGAMP. Therefore, %B/B_0_ represents the binding signal of a given sample (B) normalized to the maximum binding signal obtained in the absence of free analyte (B_0_), expressed as a percentage and calculated as follows:

$$\%B/B_{0}\frac{100\times B}{B_{0}}$$

Because it is a competitive ELISA, %B/B_0_ is inversely proportional to the concentration of free 2′3′-cGAMP present in the sample.

**B. Michaelis–Menten plot: Determination of V_max_, k_cat_, and k_1/2_ in the presence or absence of the test molecule**

**Figure 2.**
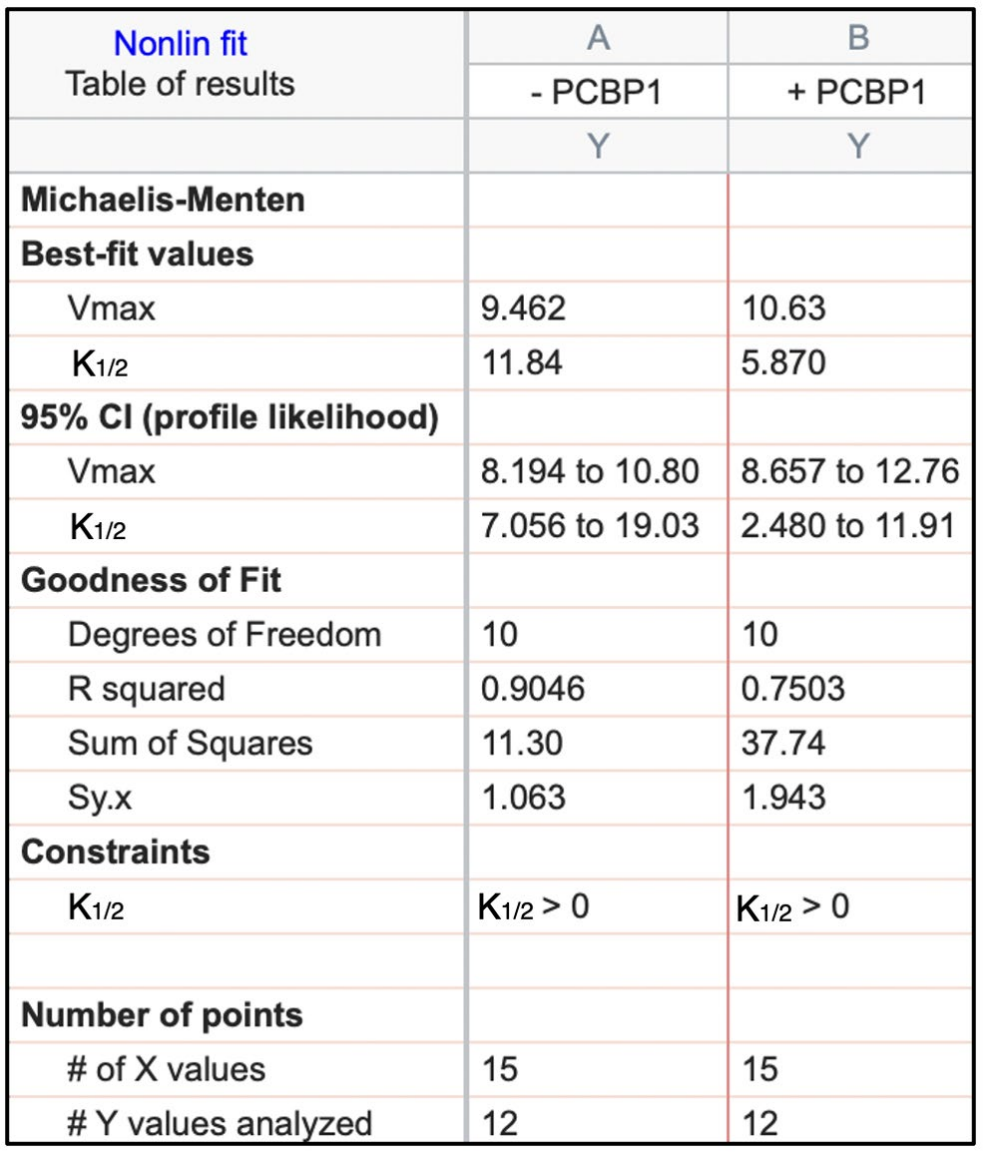
Example of Michaelis–Menten analysis generated with GraphPad Prism. The quality of the nonlinear regression was assessed using the coefficient of determination (R^2^) and 95% confidence intervals (CI) for the fitted parameters. R^2^ indicates the proportion of variance in the observed data explained by the Michaelis–Menten model, with values closer to 1.0 reflecting a stronger fit. The 95% CI provides an estimate of the precision of V_max_ and k_1/2_, with narrower intervals indicating greater reliability [8].

**Figure 3.**
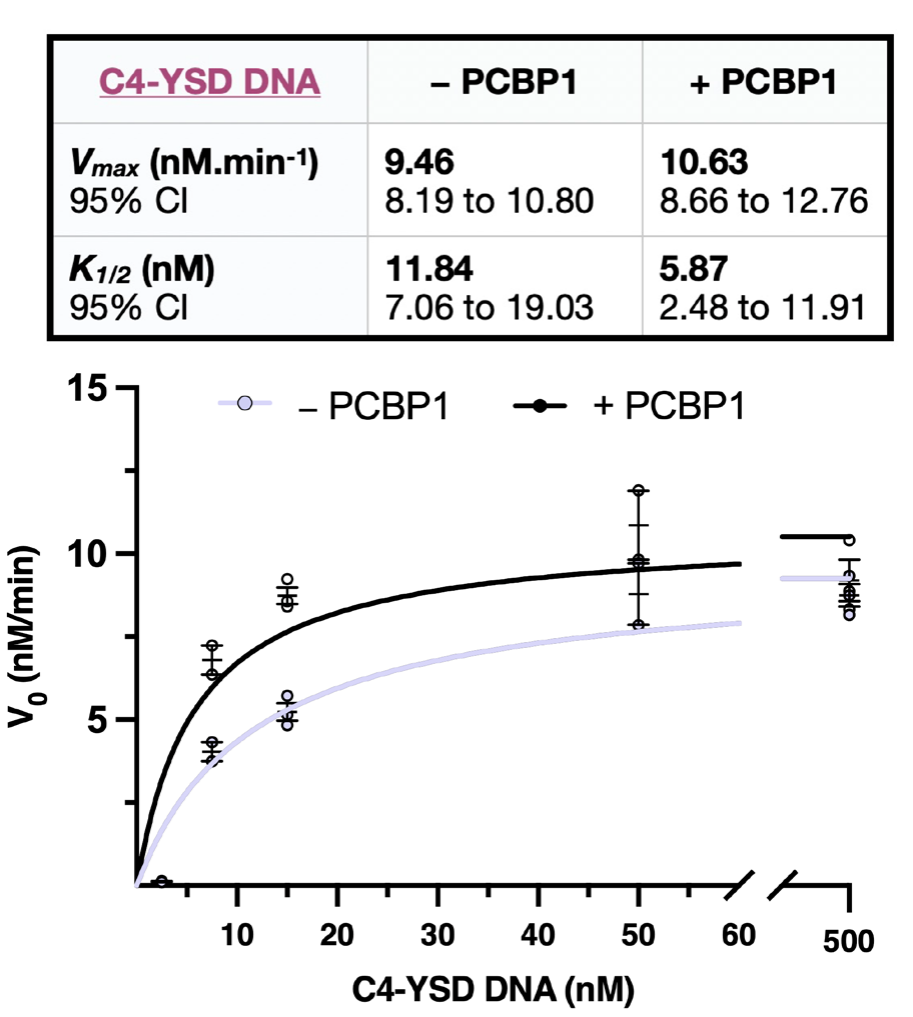
Example of a Michaelis–Menten plot generated with the data in Table 2, followed by Vmax and k_1/2_ calculations. Initial velocities (V_0_) were fitted using nonlinear regression to the Michaelis–Menten equation in GraphPad Prism. Data are presented as mean ± SEM from 2–3 independent enzymatic reactions per condition, depending on DNA concentration. Individual replicate values are shown in Table 2. Kinetic parameters were estimated with 95% confidence intervals [8].

1. After completing step B12 and the ELISA, convert the %B/B_0_ values into 2′3′-cGAMP concentrations (pg/mL) using the ELISA standard curve, then convert to nanomolar (nM). Normalize to the incubation time to obtain the rate of product formation in nM/min. For example, if x nM of 2′3′-cGAMP is produced in 30 s, multiply that value by 2 to express the rate per minute.

2. Use GraphPad Prism to create an XY table with the DNA ligand concentrations entered in the X-axis and the corresponding initial reaction rates (V_0_, in nM/min) entered in side-by-side replicate columns for the Y-axis.

3. Enter three independent replicate values for each DNA ligand concentration under both the control condition and the test condition.

4. Click *Analyze* > *Regression and Curves* > *Nonlinear Regression (Curve Fit)*. Select your dataset. Then, under the *Enzyme kinetics – Velocity as a function of substrate* drop-down menu, select *Michaelis–Menten*.

5. In the *Results* section, the V_max_ and k_1/2_ values for both the control and test conditions will be reported along with their associated 95% confidence intervals (CI) (Figure 2). Increasing the number of biological replicates will further narrow these intervals and strengthen the reliability of the parameter estimates. In the *Graphs* section, nonlinear regression curves will be shown for both the control and test conditions (Figure 3). Data points can be displayed with error bars representing either the standard deviation (SD) or the standard error of the mean (SEM). The catalytic turnover number (k_cat_) can be readily calculated using the following relationship:

$$k_{cat}=\frac{V_{max}}{{\left[E\right]}_{total}}$$

where [E]_total_ is the concentration of enzyme in the reaction.

Since the enzymatic reactions were performed with 30 nM cGAS, k_cat_ (min^-1^) can be obtained as:

$$k_{cat}=\frac{V_{max}\;(nM.{min}^{-1})}{30\;(nM)}$$

Here, k_cat_ represents the number of molecules of 2′3′-cGAMP produced per minute per molecule of cGAS, whereas V_max_ reflects the total concentration of product formed per minute by the entire 30 nM enzyme population present in the assay. A change in k_cat_ indicates a corresponding change in the intrinsic catalytic activity of cGAS, independent of enzyme concentration. Therefore, a higher k_cat_ in the presence of a test molecule would represent an increase in cGAS catalytic activity.

Lastly, the k_1/2_ value represents the DNA concentration at which the reaction rate reaches half of V_max_ and is interpreted as an indicator of cGAS’s apparent affinity for DNA. A lower k_1/2_ indicates that cGAS achieves half-maximal velocity at a lower DNA concentration, reflecting higher apparent affinity, whereas a higher k_1/2_ reflects reduced apparent affinity (see General note 3).

**Table 2.** Dataset example entered in GraphPad Prism to generate a Michaelis–Menten curve. The initial rate of reaction V_0_ obtained for each DNA ligand concentration (2.5, 7.5, 15, 50, and 500 nM) was provided in the absence (control condition) or presence (test condition) of a protein suspected to be a co-sensor of cGAS [8].

DNA ligand (nm)	V_0_ control condition (nM/min)			V_0_ test condition (nM/min)		
2.5	0.11	0.11		0.1	0.13	
7.5	4.32	3.74		6.36	7.23	
15	4.83	5.14	5.72	8.41	9.23	8.57
50	7.84	9.71		9.82	11.9	
500	8.74	8.16	9.33	8.87	8.32	10.41

## Validation of protocol

This protocol has been used and validated in the following research article (open access):

• Fréreux et al. [8]. PCBP1 Binding to Single-Stranded Poly-Cytosine Motifs Enhances cGAS Sensing and Impairs Breast Cancer Development. *Commun. Biol.*, vol. 9, 179, 2026, doi: 10.1038/s42003-025-09456-z. (Figure 7G–L and Supplementary table S3).

## General notes and troubleshooting

**General notes**

1. Replicates and batch consistency: Each test condition should always be performed in parallel with its corresponding control within the same batch (for example, ± test molecule). However, independent biological replicates or assays with different DNA ligand concentrations do not need to be carried out all at once; they can be performed on separate days, provided the cGAS enzyme has been properly aliquoted and stored at -80 °C to preserve activity.

2. This protocol is not suitable for pre-steady-state kinetics. The method relies on endpoint ELISA measurements after incubation, which cannot capture very short time scales (milliseconds to seconds). Therefore, it cannot resolve rapid pre-steady-state events such as burst phases or conformational transitions.

3. Apparent DNA-dependence constant k_1/2_: In the Michaelis–Menten model, the Michaelis constant, k_m_, represents the substrate concentration at which the reaction rate reaches half of V_max_ and is commonly interpreted as an indicator of the enzyme’s apparent affinity for its substrate. A lower k_m_ value indicates that the enzyme achieves half-maximal velocity at a lower substrate concentration, reflecting a higher apparent affinity, whereas a higher k_m_ value reflects reduced apparent affinity. However, the Michaelis–Menten model is based on the assumption that the substrate concentration needs to be in large excess over the enzyme so that the concentration of substrate bound to the enzyme, [ES], is negligible relative to the concentration of total substrate. Therefore, the kinetic parameters can be calculated using the total concentration of substrate rather than using [ES], which is unknown at low substrate concentrations. In this assay, it is sometimes necessary to use dsDNA concentrations that are close to or even below the concentration of cGAS in order to evaluate DNA-dependent activation. Under these conditions, a substantial fraction of the DNA becomes bound to cGAS, violating the requirement that substrate binding does not significantly alter the concentration used for kinetic analysis. Consequently, fitting velocity vs. total DNA yields an apparent DNA-dependence constant rather than a mechanistic k_m_. For this reason, we report this parameter as k_1/2_, defined as the total DNA concentration required to reach 50% of V_max_ under the experimental conditions. Although k_1/2_ is not a mechanistic k_m_, it reliably captures the DNA sensitivity of cGAS activation and allows comparison between control and test conditions, as the same limitation applies uniformly across all curves. In addition, because dsDNA functions as an allosteric activator of cGAS rather than the true chemical substrates (ATP and GTP), the term k_1/2_ is conceptually more appropriate for describing ligand-dependent activation. Finally, V_max_ is not affected by this limitation because, at high DNA concentrations where saturation is achieved, the condition [S]_free _≈ [S]_total_ becomes valid again. At these saturating ligand concentrations, the reaction rate reflects *k*_cat_ × [*E*]_total_, ensuring that V_max_ can be reliably determined in this experimental format.

4. Limited ELISA sensitivity: Because this assay relies on an endpoint ELISA with a limited dynamic range and sensitivity, it may be relatively insensitive to small changes in 2′3′-cGAMP concentration. Low-activity cGAS variants, weak DNA activators, or subtle inhibitor effects may produce cGAMP levels close to the detection limit of the assay. Under such conditions, differences in catalytic output may be underestimated or not reliably detected.

5. Potential interference with ELISA detection: Small molecules, proteins, nucleic acids, or buffer components added to the reaction mixture may interfere with the ELISA detection system itself. For example, a test compound could bind assay antibodies, mask the 2′3′-cGAMP epitope, alter antibody–antigen interactions, or affect signal development. In such cases, an apparent change in measured cGAS activity may reflect assay interference rather than a true effect on enzymatic catalysis. To control for this possibility, defined concentrations of 2′3′-cGAMP standards should be spiked in the presence of the test molecule to verify that ELISA performance and standard curve characteristics remain unaffected.

**Troubleshooting**

**Problem 1:** High 2′3′-cGAMP ELISA absorbance values (comparable to B_0_ values).

Possible cause: Insufficient 2′3′-cGAMP production.

Solutions: Include all recommended ELISA controls (blank, NSB, B_0_, TA) to confirm proper assay performance. Verify that the cGAS enzyme was correctly aliquoted and stored at -80 °C; do not refreeze or reuse aliquots. If necessary, increase the cGAS concentration per reaction (e.g., 60 nM instead of 30 nM).

**Problem 2:** Low 2′3′-cGAMP ELISA absorbance values.

Possible cause: Excessive accumulation of 2′3′-cGAMP beyond the linear range of the ELISA.

Solutions: Shorten the reaction time, ensuring it remains within the validated linear range of product formation. Alternatively, decrease the cGAS concentration per reaction.

**Problem 3:** Inconsistent replicate values.

Possible cause: Variability in cGAS enzyme activity or handling.

Solutions: Confirm that the cGAS enzyme was properly aliquoted and stored at -80 °C; never reuse the same aliquot. Use the same batch of recombinant cGAS enzyme for all the kinetics if possible. Prepare reaction master mixes (step B5) to minimize pipetting variability between control and test conditions. Use consistent and precise incubation times across all conditions and batches. Increase the number of replicates to improve reproducibility and narrow confidence intervals for kinetic parameter estimates.
